# Role of breeding and natal movements in lifetime dispersal of a forest‐dwelling rodent

**DOI:** 10.1002/ece3.2814

**Published:** 2017-03-02

**Authors:** Vesa Selonen, Ralf Wistbacka

**Affiliations:** ^1^ Section of EcologyDepartment of BiologyUniversity of TurkuTurkuFinland; ^2^ Department of BiologyUniversity of OuluOuluFinland

**Keywords:** bequeathal, gene flow, movement ecology, population spread

## Abstract

The lifetime movements of an individual determine the gene flow and invasion potential of the species. However, sex dependence of dispersal and selective pressures driving dispersal have gained much more attention than dispersal at different life and age stages. Natal dispersal is more common than dispersal between breeding attempts, but breeding dispersal may be promoted by resource availability and competition. Here, we utilize mark–recapture data on the nest‐box population of Siberian flying squirrels to analyze lifetime dispersal patterns. Natal dispersal means the distance between the natal nest and the nest used the following year, whereas breeding movements refer to the nest site changes between breeding attempts. The movement distances observed here were comparable to distances reported earlier from radio‐telemetry studies. Breeding movements did not contribute to lifetime dispersal distance and were not related to variation in food abundance or habitat patch size. Breeding movements of males were negatively, albeit not strongly, related to male population size. In females, breeding movement activity was low and was not related to previous breeding success or to competition between females for territories. Natal philopatry was linked to apparent death of a mother; that is, we did not find evidence for mothers bequeathing territories for offspring, like observed in some other rodent species. Our results give an example of a species in which breeding movements are not driven by environmental variability or nest site quality. Different evolutionary forces often operate in natal and breeding movements, and our study supports the view that juveniles are responsible for redistributing individuals within and between populations. This emphasizes the importance of knowledge on natal dispersal, if we want to understand consequences of movement ecology of the species at the population level.

## 1 Introduction

The dispersal may consist of movements at different life and age stages of an individual. Dispersal behavior at different life stages has, however, gained much less attention than sex dependence of dispersal or general selective pressures potentially driving dispersal (Berteaux & Boutin, 2000; Starrfelt & Kokko, 2012). It is known that natal dispersal, the movement away from birthplace before the first breeding, is generally more common than breeding dispersal, which occurs between two breeding attempts (Greenwood & Harvey, 1982; Johst & Brandl, 1999; Paradis, Baillie, Sutherland, & Gregory, 1998). However, studies on breeding dispersal concentrate on certain species groups, such as birds (Paradis et al., 1998), while, for example, in small‐ and medium‐sized mammals, these studies are scarce (Berteaux & Boutin, 2000; Le Galliard, Remy, Ims, & Lambin, 2012). For a comprehensive understanding of dispersal, we need knowledge on movements at different ages and life stages, because it is the lifetime movements of an individual that determine the gene flow and the invasion potential of a species (Ronce, 2007).

The major difference between natal and breeding dispersal is that a juvenile needs to locate its first breeding site, whereas an adult has already located at least one possible breeding site. Several factors may still promote breeding dispersal, including breeding success in the past and, in territorial species, the possession and quality of breeding territory (Arlt & Pärt, 2008; Johst & Brandl, 1999; Öst, Lehikonen, & Jaatinen, 2011; Switzer, 1993). Some species may pay attention to both intrinsic site quality and their own breeding success (Kokko, Harris, & Wanless, 2004). In addition, temporal variation in the amount of food can promote breeding dispersal (Wauters, Lens, & Dhondt, 1995). All these results imply that breeders can be sensitive to current site quality. Breeding dispersal, however, imposes the risk of losing resources that an individual already has in its possession (Danchin & Cam, 2002; Harts, Jaatinen, & Kokko, 2016; Morris, 1982), but misjudgments on quality of site of living or territory as well as variation in habitat quality over time and space can result in a situation where an individual's possession turns into an asset not worth protecting (Krebs, 1971; Mestre & Bonte, 2012; Wauters et al., 1995).

An interesting phenomenon possibly explaining breeding dispersal in rodents is the bequeathal of territories for offspring (Cockburn, 1988). For example, regarding the North American red squirrel, *Tamiasciurus hudsonicus*, juveniles moving off the natal territory incur survival costs (Berteaux & Boutin, 2000; Boutin, Larsen, & Berteaux, 2000). To increase the fitness of both the juveniles and the mothers, about 15% of red squirrel mothers were observed to give up part or all of their territory to their offspring. This behavior is suggested to represent a form of parental investment (Berteaux & Boutin, 2000; Boutin et al., 2000). However, it remains uncertain how general this type of bequeathal behavior is in rodents (Lambin, 1997; Wauters et al., 1995). Lambin (1997) in his review found support for bequeathal hypothesis from for three rodent species (red squirrels, Columbian ground squirrels, and kangaroo rats).

**Figure 1:**
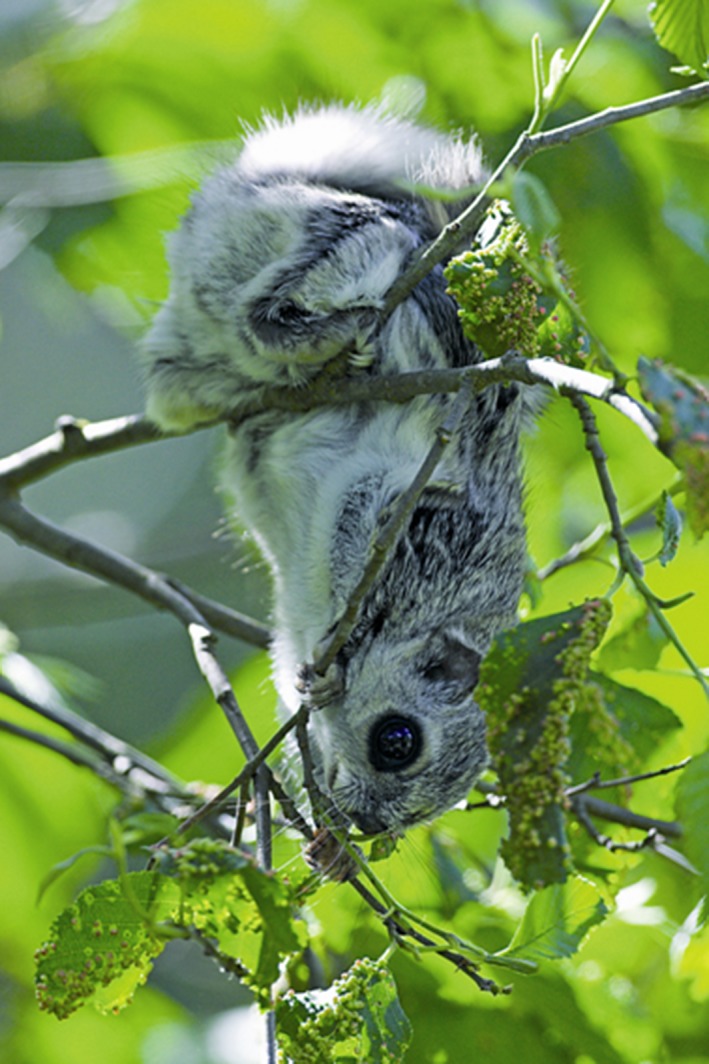
The study organism, Siberian flying squirrel *Pteromys volans*. Photograph by Henrik Lund

Here, we utilize long‐term mark–recapture data to study natal and breeding dispersal patterns of an arboreal rodent, the Siberian flying squirrel, *Pteromys volans* (Figure 1). Flying squirrels were monitored in two nest‐box populations from 1993 to 2014 in western Finland. By definition dispersal, both natal and breeding, is a permanent change in a home‐range location (Clobert, Baguette, Benton, & Bullock, 2012). We analyze changes in location of nest sites from natal nest to breeding time nest sites during the individual lifetime. We define distance between natal nest and first breeding time nest as “natal dispersal.” Adulthood nest site changes between breeding attempts are from now on called “breeding movements.” We did not study permanent changes in home‐range location, and breeding movements do not necessarily indicate “breeding dispersal.” However, we use the data for breeding movements to discuss possible role of breeding dispersal in flying squirrels. The natal dispersal of the flying squirrel has been intensively studied with radio‐telemetry methods (e.g., Hanski & Selonen, 2009; Selonen & Hanski, 2010, 2012); thus, we can compare movements observed here to those obtained by radio telemetry. Based on the radio‐telemetry studies, natal dispersal is female‐biased in flying squirrels. The long female dispersal distances are likely related to competition between females for territories (Hanski & Selonen, 2009). In general, social factors and territory availability are important determinants of dispersal in arboreal squirrels (Boutin, Tooze, & Price, 1993; Wauters et al., 1995). Territory availability may affect breeding movements of females, but males may need to move longer distances when mate availability decreases (Selonen, Painter, Rantala, & Hanski, 2013). The flying squirrel is a forest specialist, and cavities or nest boxes are key elements in flying squirrel territories (Selonen & Hanski, 2012). Thus, similar to above‐mentioned rodent species observed to bequeath territories to offspring (Lambin, 1997), flying squirrels juveniles need to locate a resource that is essential for their future fitness (Selonen & Hanski, 2012).

We specifically aim to study the lifetime dispersal pattern of individuals and compared breeding movements to natal dispersal distances, in an aim to evaluate the possible contribution of breeding movements to gene flow and population redistribution. We hypothesized that poor environmental conditions and high population density drive breeding movements. For females, we hypothesized that low breeding success in the previous year would increase their likelihood to abandon the nest site. Keeping in mind that female flying squirrels seem to be territorial (Selonen et al., 2013), if competition between females determines breeding movements, we hypothesized that changes in nest site are related to colonization of another female. Finally, we sought evidence for bequeathal of territories for offspring, that is, cases where a mother has moved to another site and her offspring have remained philopatric within their natal territory. As an alternative hypothesis to bequeathal, we analyzed whether natal philopatry is related to the apparent death of the mother.

## 2 Material and Methods

### 2.1 Study species

The Siberian flying squirrel is a nocturnal, arboreal rodent, which lives in spruce‐dominated boreal forests. The flying squirrel feeds in deciduous trees that occur within spruce forests, and birch and alder catkins are the species’ main food sources. Flying squirrels also cache alder catkins for forage during winter and early spring (Mäkelä, 1996; Selonen, Wistbacka, & Korpimäki, 2016). The mating season of flying squirrels starts in mid‐March. After the first litter is born in April, the female flying squirrel can have a second litter in June. The juveniles disperse in autumn (Hanski & Selonen, 2009). The mating system is promiscuous with elements of scramble competition between males with multiple paternities within litters (Selonen, Wistbacka, & Santangeli, 2016; Selonen et al., 2013). Sex ratio appears even, and individuals that survive juvenile stage live on average 2 years in total with maximum observed 7 years (own observation). Females seem to be territorial, living in separate home ranges, but males live in overlapping home ranges, which encompass several males and females (Selonen et al., 2013). Outside the breeding season, males and females often nest communally, although in most cases, perhaps related to the low density of individuals, flying squirrels have been observed to be solitary in their nests (Selonen, Hanski, & Wistbacka, 2014). Individuals use several nests within a year, in tree cavities, nest boxes, and dreys (twig nests), but the latter are not preferred for breeding, and cavities are a limiting resource in managed forests (Selonen & Hanski, 2012).

### 2.2 Study areas

The studies on flying squirrels were carried out in two areas: Luoto (63°49′N, 22°49′E) and Vaasa (63°3′N, 22°41′E). In Luoto, flying squirrels were studied from 1993 to 2014 within an area of 44 km^2^. The main forest types in Luoto are shoreline spruce‐dominated mixed forests, clear‐cuts, and cultivated Scots pine forests. The Vaasa study area is located about 100 km southwest of Luoto. The marking of flying squirrels started in 1992 in the central part of Vaasa (3.8 km^2^). The area was extended in 2,000 to cover 40 km^2^. Vaasa is covered by spruce forest patches, clear‐cut areas, and agricultural fields (for more information, see Lampila, Wistbacka, Mäkelä, & Orell, 2009; Selonen et al., 2014). The landscape structure is patchy because of forest management, and life of flying squirrels is concentrated within forest patches (Selonen, Hanski, & Stevens, 2001).

Nest boxes for flying squirrels were placed in forest patches of various sizes in sets of two to four nest boxes per site, on average two nest boxes per forest hectare. Flying squirrels were captured by hand in nest boxes, sexed, weighed, and marked with ear tags (Hauptner 73850, Hauptner, Germany). The nest boxes had entrance‐hole diameters of 4.5 cm, the same size as the cavity entrances made by the great spotted woodpecker, *Dendrocopos major*, which represent the most common natural nesting site for flying squirrels in our study area. The nest boxes were made from a piece of aspen or spruce trunk, so that they resembled natural cavities. Based on earlier analyses, the recapture probability was high, around 70–80% (Brommer, Wistbacka, & Selonen, 2017; Lampila et al., 2009), as natural cavities were rare in our study areas (for differences, or lack thereof, in the use of natural cavities versus our nest boxes, see Selonen et al., 2014). Thus, disappearance of an individual indicated apparent death. Nest boxes were checked in June each year. Occupied sites were again checked in August because flying squirrel females can have two broods per year. Sporadic nest box checks occurred also during other seasons (for more information, see Selonen et al., 2014; Brommer et al., 2017). The June and August sessions covered the first and second breeding attempts of the year. In most cases, we observed a litter of small juveniles with their mother in the nest boxes, but in some cases, large juveniles were observed without their mother. These individuals were classified as predispersal juveniles, if they were smaller than 70 g when observed. Larger juveniles were omitted from the data as we could not be certain about their birth site. Juveniles weighing <70 g are still strictly dependent on the natal nest (Hanski & Selonen, 2009).

### 2.3 Data gathering

#### 2.3.1 Natal and breeding dispersal

Natal dispersal was the distance between the natal nest and the nest where an individual was located when first seen after natal dispersal period. Natal dispersal occurs during the first autumn after birth, based on radio‐telemetry studies (Hanski & Selonen, 2009); that is, in our data, the first observation after natal dispersal was in summer when individuals were 1 year old. We used the Vaasa study area data for calculating natal dispersal distances only after the year 2,000, as before that, the studied area was too small for detecting long‐distance dispersal. However, data from the entire study period in Vaasa were used for breeding movement analysis, as distances moved did not differ before and after the year 2,000 in this area (males: *F*_55.1_ = 2.8, *p* = .11; females: *F*_53_ = 0.1, *p* = .89). In regard to the Luoto study area, the entire study period, 1993–2014, was used.

Adulthood breeding movements were the nest site changes occurring after the first breeding attempt (second year of life and following years). Data consisted of observations from one to six separate years per individual (average 2.6 ± 1.1 SD). There was often more than one observation per year (see Section 3), as nest boxes were checked twice a year. These observations were between spring and summer breeding attempts, and thus, there was potential for breeding dispersal to have occurred. In addition, sometimes an individual was not located every year (54 observations of 1,090 breeding moves). Thus, the length of time period between two observations might vary, and this was taken into account in the analysis.

Limits of the study area likely biased observations toward short movements, because long‐distance dispersers may have moved outside the mark–recapture area. However, observed natal dispersal distances in our study (see Section 3) were quite similar to those reported in radio‐telemetry studies (2.4 km for females and 1.1 km for males, Hanski & Selonen, 2009). The reason for this is that the extent of our study areas was exceptionally large (the size and shape of study areas allow observing distances up to 10–11 km) and both study areas are located along the coastline (Luoto is actually a peninsula). Furthermore, our study areas were surrounded by fields; thus, both the fields and the sea decreased individual movements outside the study area (for figure of our nest‐box study area, see Selonen et al., 2014). Nevertheless, it is obviously possible that some individuals moved outside the study area, and to control for the effect of the edge of the study area on observed movement distances, and also variation in nest‐box availability within the study area, we calculated the number of nest boxes in a 500‐m buffer around each nest box.

Foraging movements of flying squirrels concentrate in the surroundings of the nest site (on average within 100 m from nest site, Hanski, Stevens, Ihalempiä, & Selonen, 2000), and the edges of male home ranges are determined by locations of female nest sites (Selonen et al., 2001). Further, individuals tolerate each other in the same nest: Females often nest with males, except during breeding season, and a male may nest with other males (Selonen et al., 2014). Thus, the nest site locations give a good approximation of movement patterns of individuals (see also Section 4). In addition, natural cavities are rare in our study areas (0.1 per spruce forest hectare based on 742 hectares surveyed within our study areas), which increases the likelihood of locating individuals from nest boxes. Nevertheless, it is clear that with the method used, mark–recapture based on flying squirrels residing in nest boxes, we could observe only part of the movements performed by the study individuals. Moreover, the individuals that did not locate nest sites were not included in the analysis. However, the survival of these individuals is questionable (Berteaux & Boutin, 2000; Boutin et al., 2000).

#### 2.3.2 Environmental factors affecting breeding movements

We analyzed the effect of yearly changes in food abundance on observed breeding movements of flying squirrels. Based on previous analysis, yearly variation in alder catkin production is an important determinant of flying squirrel reproduction (Selonen & Wistbacka, 2016; Selonen, Wistbacka, & Korpimäki, 2016). We also tested the effect of birch catkins but left it out from current analysis, as alder appears to be a more important food resource than birch for reproduction of flying squirrels (Selonen & Wistbacka, 2016). Following Selonen, Wistbacka, & Korpimäki (2016), we used aerial pollen estimates that correlate with catkin production (Ranta et al., 2008) as a proxy for yearly alder catkin production. Pollen data were collected by the aerobiology unit at the University of Turku. For both study areas, we used the estimate for central western Finland, which was sampled in Vaasa. This estimate does not control for spatial variation in catkin production within our study areas, but catkin production is spatially correlated at scales of up to a few hundred kilometers in Finland (Ranta et al., 2008). Thus, there should not be any major variation in catkin production within our study areas. In addition, to account for territory‐level differences in catkin production, we categorized each nest‐box site into three categories: sites where alders were rare, sites with an average amount of alders, and sites with many alders. The alder estimate for each site was evaluated during field visits.

We calculated the forest patch size around each nest box. In cases where an individual changed patches, the size of the patch that the individual moved out of was used in the analysis. Forest patches were mapped using aerial photographs (for more information, see, for example, Selonen & Hanski, 2012). Forest cuttings affected flying squirrels because the study individuals were living in actively managed forests (Santangeli, Wistbacka, Hanski, & Laaksonen, 2013; Selonen & Hanski, 2012). However, the cases where a flying squirrel was present before and after cutting were uncommon (29 cases with cuttings), and the occurrence of forest cuttings, a binomial variable, either being present or not present in the site, did not affect the observed breeding movements (effect on the observed breeding movements of sexes combined: *F*_1,967.8_ = 2.06, *p* = .15). Even so, cuttings obviously influenced forest patch sizes in the study area. Information on forest cuttings was based on landscape analysis obtained from Landsat satellite images and aerial photographs. Finally, we calculated number of males and females located from nest boxes within the study areas each year (from now on population size of males and females).

### 2.4 Analyses

#### 2.4.1 Breeding movements and environmental variables

To analyze environmental factors explaining breeding movements, we performed generalized linear mixed models (glmm) with lognormal distribution in Glimmix, SAS (9.3). Males (461 observations for 166 males) and females (630 observations for 232 females) were modeled separately. The breeding movement distance (distance between nest sites after the natal dispersal period) was used as a dependent variable. A model included the following variables: study area (Vaasa or Luoto), length of time period between observations (between spring and summer breeding or between breedings of consecutive years), number of available nest boxes within 500 m of each nest box (continuous variable), and individual ID as a random variable using Kenward–Roger determination of degrees of freedom. These variables were included in all models. Instead, from environmental variables, we dropped the nonsignificant variables from the further models. The environmental variables were abundance of alder trees at each nest site (categorical: few, average, high), the alder pollen estimate for each year (continuous), forest patch size (continuous variable), and male or female population size (continuous). In the end, these variables were analyzed in separate models (the results remained the same if all environmental variables were included in the same full model), because all variables except male population size remained nonsignificant (see Section 3; male population size was included in all male models). In addition, due to obvious correlation between male and female population size, these two variables were not included in the same model. In models including population size, we also included the total number of nest boxes checked each year. Variables included in the models and their average values can be seen in Table 1.

**Table 1:** Results from analyses explaining breeding movements in male and female flying squirrels

	*n* or average ± SD^a^	Estimate	*F*	*df*	*p*
Males					
Study area	Luoto = 208, Vaasa = 253	Luoto: −0.73 ± 0.39	3.5	1,121.4	.06
Alder year	950 ± 820 pollen/1 m^3^ of air	−0.0001 ± 0.0002	0.34	1,416.1	.56
Alder site^b^	1 = 116, 2 = 177, 3 = 167	1: 0.54 ± 0.34, 2: 0.27 ± 0.3	1.22	2,274.2	.3
Available nest boxes^c^	13 ± 6 boxes	−0.007 ± 0.02	0.13	1,263.1	.71
Female population size	14 ± 7 individuals	−0.004 ± 0.03	0.02	1,309	.88
Male population size	15 ± 9 individuals	−0.08 ± 0.03	5.4	1,325.2	**.02**
Forest patch size	3.6 ± 2.2 ha	−0.05 ± 0.08	0.5	1,291.9	.48
Gap^d^	0 = 212, 1 = 214	0: 0.19 ± 0.23,	0.76	1,394.9	.38
Females					
Study area	Luoto = 248, Vaasa = 382	Luoto: −0.15 ± 0.4	0.15	1,298.7	.69
Alder year	950 ± 820 pollen/1 m^3^ of air	0.0001 ± 0.00001	0.75	1,586	.39
Alder site^b^	1 = 100, 2 = 269, 3 = 261	1: 0.5 ± 0.36, 2: 0.4 ± 0.27	1.4	2,300.8	.25
Available nest boxes^c^	12 ± 6 boxes	0.09 ± 0.02	23.7	1,327.1	**<.0001**
Female population size	15 ± 6 individuals	0.02 ± 0.03	0.84	1,422.7	.36
Male population size	14 ± 9 individuals	0.01 ± 0.03	0.26	1,431.7	.61
Forest patch size	3.9 ± 2.5 ha	−0.004 ± 0.05	0.01	1,275	.93
Gap^d^	0 = 283, 1 = 328	0: −0.05 ± 0.16	0.12	1,489.9	.72
Offspring no. in previous year^e^	2.2 ± 1.5 juveniles	0.07 ± 0.09	0.71	1,321.9	.4

a Number of cases for 166 males and 232 females or average values for estimates between 1992 and 2014.

b 1 = alder rare in the nest‐box site, 2 = average amount of alders, 3 = alder abundant.

c Within a 500‐m buffer.

d 0 = the observations were between first and second breeding attempts within 1 year, 1 = one year between observations, that is, between breeding attempts of consecutive years. In cases where there was a gap longer than 1 year between observations (54 cases), those observations were omitted from the analyses.

e Separate analysis (*n* = 341 moves for 179 females). For model structure of analysis for environmental variables, see Section 2 (estimates for many variables are from separate models).

Bold values to indicate significant results.

We analyzed also whether or not the distance of breeding movements was correlated with natal dispersal distance. The model included as explanatory variables natal dispersal distance, study area, length of time period between observations, number of available nest boxes, and individual ID as a random variable. For this analysis, we had 77 males (225 moves) and 88 females (247 moves) with data on both natal and breeding movement distance. The number of individuals with data for only natal dispersal distance was 147 males and 154 females.

#### 2.4.2 Role of breeding movements on lifetime dispersal

We built a model where the straight‐line distance from the natal nest at the time of observation was the dependent variable. This could be performed for individuals ear‐tagged as juvenile and observed more than once as adult and, thus, had data for breeding movements (above‐mentioned 77 males and 88 females). Thus, we could analyze whether or not breeding movements contributed to the lifetime dispersal distance of individuals. Among the explanatory variables, we included the year of breeding observation (year 1, year 2, year 3…). Sexes were combined for this analysis, and sex was included as a class variable in the model. We also tested the effect of the interaction term between sex and the year of breeding observation. The study area was used as a class variable and individual ID as a random variable (with Kenward–Roger determination of degrees of freedom).

#### 2.4.3 Females and territoriality

For adult females with data on the breeding success of the previous year and data for breeding movements (*n* = 179 females; for more information on our litter data, see Selonen, Wistbacka, & Korpimäki, 2016), we looked for a correlation between the number of offspring produced during the previous year and breeding movements of the subsequent year (dependent variable). Moves between June and August capturing sessions were omitted from this analysis; that is, only moves between June/August of the previous year and the subsequent June were included. In addition, for cases where an adult female changed nest box, we checked to see whether the previous nest box was occupied by another individual. This was performed for females only, as males are less territorial than females (Selonen et al., 2013). Breeding movements (dependent) were related to the class variable whether or not the nest box abandoned by a female was in use by another female or male. The models included study area, length of time period between observations, number of available nest boxes, and individual ID as a random variable.

#### 2.4.4 Bequeathal of nest sites for offspring

To analyze bequeathal, first, we compared the breeding movements of females with and without philopatric juveniles. Bequeathal could be indicated, if the mother with philopatric juveniles moved further away from the nest than females without philopatric juveniles moved between two breeding attempts (dependent variable distance moved by mother; class variable for female having or not philopatric juvenile included in the model). A philopatric juvenile was an individual that was observed within natal nest‐box site after maturation, that is, after autumn of first living year. Second, we performed a model where the mother's presence (located in the following year vs. not located) was the dependent variable (binomial distribution) and the juveniles’ natal dispersal distance was the explanatory variable. With this model, we could analyze whether the absence of the mother was related to the juveniles’ philopatry. The study area and the sex of the juvenile were included as class variables, and the nest‐box site (forest patch where the nest box was located) was included as a random variable.

## 3 Results

### 3.1 Natal dispersal and breeding movements

**Figure 2:**
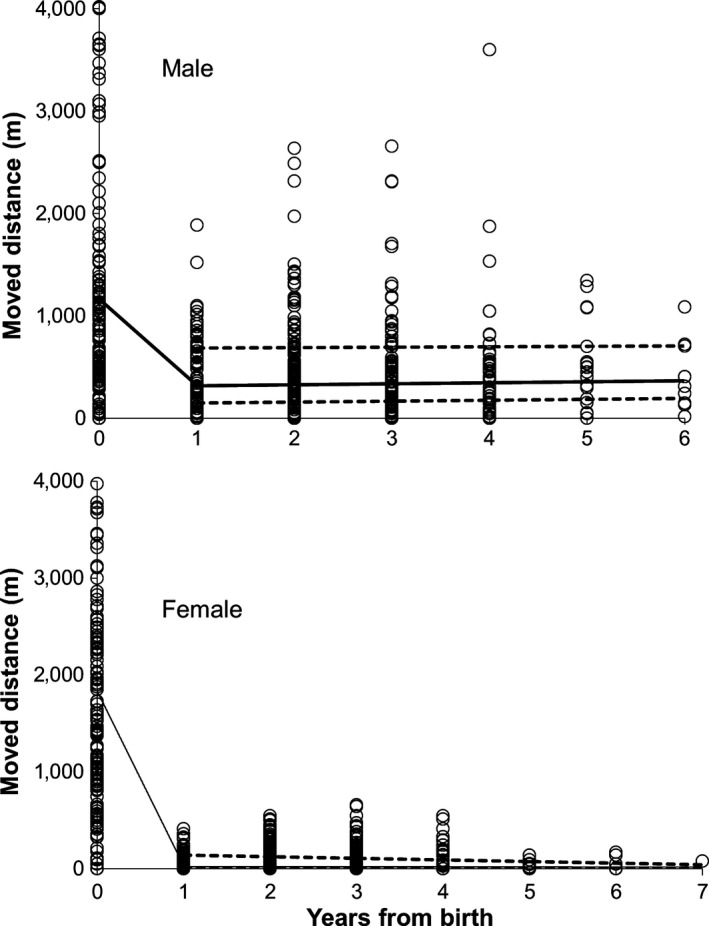
Lifetime movement activity in male and female flying squirrels: Year 0 indicates distance moved before first breeding (natal dispersal), year 1 indicates breeding moves observed within first breeding summer, and for years 2–7, breeding movements within a year not shown. Line for breeding movements is based on predicted mean values and dashed line for upper and lower confidence limits. There is no indication of a nonlinear relationship for years 1–7 (interaction between breeding movement distance and year; *p* > .05 for both sexes). The model for breeding movements (years 1–7) was not used to model natal dispersal, and figures also include the cases with only natal or breeding observations. Thus, confidence limits are lacking between 0 and 1 (difference between average values of 0 and 1–7: *p* < .0001 for both sexes). Circles are raw data. *Y*‐axis limited to 4 km for visibility; *n* = 8 females and 6 males had dispersal distance over 4 km, maximum observed 6.7 km

For individuals tagged as juveniles, observed average (±SD) natal dispersal distances were 1,830 ± 1,390 m and 1,200 ± 1,340 m for females (*n* = 154) and males (*n* = 147), respectively (difference between sexes: *F*_1,298_ = 19, *p* < .0001). After the natal dispersal period, movement activity of both sexes declined, but the decline was clearer for females than for males (Figure 2). For adult females, breeding time nest site changes were on average only a few tens of meters (model predicted: 20 m; in raw data: 72 ± 130 m) with maximum of around 500–600 m between two consecutive years (Figure 2). However, there was one long‐distance move of 1,240 m (between two observations within the same summer). For males, breeding movements were around 300 m (predicted value; raw data 440 ± 430 m) with a maximum of around 2–2.5 km (difference between sexes in breeding movement distance: male = 166, female = 232; *F*_1,331.2_ = 162, *p* < .0001). Seven percent (31 of 449 cases) of breeding movements of adult males were longer than the average observed natal dispersal distances (1,200 m).

**Figure 3:**
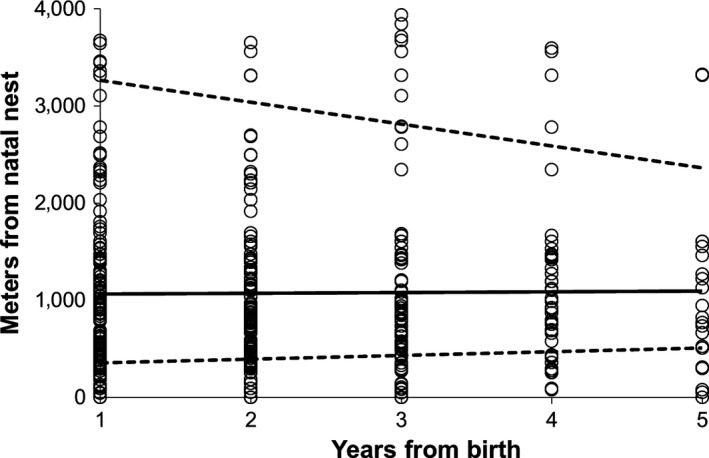
The distance from natal nest during breeding movements is compared against timing of observation in years. Individuals with data for both natal nest and for at least one breeding move are included (*n* = 165 individuals with 472 moves). Sexes are combined, as the response did not differ between sexes (interaction between sex and year: *F*_1,339.3_ = 0.2, *p* = .66). The solid line represents the predicted mean values, and the dashed line represents the upper and lower confidence limits. Circles are raw data. Movement observations within year not shown

Breeding movements did not increase or decrease the distance the individual was located from the natal nest (Figure 3). That is, movements after natal dispersal did not affect the lifetime dispersal distance of the individual (*n* = 165 individuals, distance from natal nest correlated with year of observation: estimate −0.01 ± 0.06; *F*_1,341.9_ = 0.11, *p* = .74; Figure 3). Breeding movement distance did not correlate with natal dispersal distance (77 males: estimate 0.016 ± 0.09, *F*_1,213.7_ = 0.03, *p* = .86; 88 females: estimate −0.07 ± 0.06, *F*_1,175.4_ = 1.4, *p* = .24).

### 3.2 Factors explaining breeding movements

Environmental variables describing habitat quality and food abundance were not related to breeding movements of flying squirrels (Table 1). The only variable influencing male breeding movements was male population size, as breeding movements slightly shortened when male population size increased (Table 1). For females, we did not make the same observation (Table 1). Availability of nest boxes affected observed distance of breeding movements in females, but not in males (Table 1).

For females that changed nest boxes between breeding attempts, the abandoned nest box remained nonused by flying squirrels in 276 cases, was occupied by another female in 14 cases, and was occupied by a male in 34 cases. This occupancy status of the nest box was not related to distance moved by the female who left the nest box (*F*_3,308.4_ = 0.66, *p* = .57). For females who changed forest patches between breeding attempts, the size of the new patch did not differ from the size of the original patch (difference between patches in size = −0.1 ha, compared to expected difference of 0 ha: *n* = 105 cases, *t*_104_ = −0.26, *p* = .79). Finally, breeding success, that is, the number of produced offspring during the previous year, was not related to breeding movements of females (Table 1).

### 3.3 Bequeathal of nest sites

Natal philopatry was linked to the apparent death of the mother; that is, dispersal distance was shorter when the mother disappeared (Table 2). In other words, we did not observe cases of bequeathal: There were only eight cases where a mature individual remained within its natal site and its mother was observed alive. In these cases, the observed movements of the mother did not differ from movement of females without philopatric juveniles (philopatry cases: 75 ± 118 m; no philopatry: 72 m ± 131 m; *F*_1,604.8_ = 0.05, *p* = .83). Natal philopatry was more common in males than in females (Table 2; 21% of males and 7% of females stayed within the natal site).

**Table 2:** Effect of disappearance of mother, that is, apparent death, on philopatry in flying squirrel juveniles

	Philopatric^a^	Dispersing^a^	Test for effect of presence of a mother on natal dispersal distance^c^				
	Mother present vs. not present^b^	Mother present vs. not present^b^	Variable	Estimate	*F*	*df*	*p*
Males	6 and 22 individuals	62 and 42 individuals	Dispersal distance	0.6 ± 0.16	13.5	1,205	.0003
Females	2 and 9 individuals	81 and 59 individuals	Sex	0.05 ± 0.29 (male)	0.031	1,45	.87

a Individuals divided into philopatric or dispersing based on a 200 m cutline distance, which indicates whether or not individual was within natal home range.

b Whether or not mother was located in the next or following years.

c General linear mixed model for whether or not mother was observed alive or disappeared when offspring was adult (binomial variable).

## 4 Discussion

Breeding movements did not contribute to dispersal distance and were not driven by environmental factors in flying squirrels. Breeding movements in males were related to male population size, but breeding movement activity of females was low and was not related to competition between females for territories. Natal philopatry was linked to vacancy of territories by the apparent death of the mother; that is, female flying squirrels did not bequeath natal territories for offspring.

The age‐specific movement pattern changed from female‐biased natal dispersal (see also Hanski & Selonen, 2009) to male‐biased breeding movements in the flying squirrel. This is not a general trend observed in mammals, but not surprising as various evolutionary and environmental forces likely operate in natal and breeding movements (Greenwood, 1980). For example, in arvicoline voles, breeding movement appears to be more strongly male‐biased than natal dispersal (Le Galliard et al., 2012). In flying squirrels, competition between females for territories possibly leads females to move longer distances than males during natal dispersal (Hanski & Selonen, 2009), but after natal dispersal, females stuck to the selected breeding site. Instead, movements of adult males in polygynous mammals are related to competition for mating opportunities (Andersson, 1994; Greenwood, 1980), which raises the breeding movement activity higher than that of females. Thus, males potentially contribute to gene flow more than females during adult life. However, in this study, the breeding movements of flying squirrels were centered on the location where individuals ended up during natal dispersal. That is, breeding movements did not increase or decrease the distance of the nest site used in adulthood from the natal nest. It is clear that males move around the nest site (the observation unit in this study) and that these movements likely have yearly variation. However, these movements should occur with the same probability to all directions (see e.g., Selonen et al., 2001, 2013), both toward and farther away from the natal site. In other words, our results indicate that, on average, the spread of individuals and genes across the landscape does not continue during breeding movements in flying squirrels. This is an important result on the age‐specific movement patterns of the species and highlights that lifetime dispersal is determined during the short period of natal dispersal.

One reason for the lack of clear breeding dispersal in female flying squirrels may be their dependence on cavities (or nest boxes) as nesting resources. Cavities occur sparsely in the landscape (e.g., Selonen & Hanski, 2012) and may limit the possibility for territory shifts. During the lifetime of a flying squirrel, the destruction of the nest cavity is rare, although forest management nowadays sometimes causes it to happen. Flying squirrels are also likely dominant in competition for cavities against most other cavity users in boreal forests, as the size of the cavity/nest‐box entrance hole prevents larger animals from entering the cavity. Indeed, a theoretical study predicts that low territory turnover selects for low breeding dispersal propensity (Harts et al., 2016). In contrast, female European red squirrels, *Sciurus vulgaris*, in territories with poor food resources may shift to territories with more food to improve their reproductive rate (Lurz, Garson, & Wauters, 1997; Wauters et al., 1995). The spatiotemporal variation in food abundance is quite comparable between flying squirrels and red squirrels (Selonen, Wistbacka, & Korpimäki, 2016), but apparently variation in food levels is not such a factor that flying squirrel females would abandon the nest site during poor food years. In addition, low breeding success did not explain breeding dispersal in female flying squirrels. In birds, previous breeding success has been observed to affect breeding dispersal (Arlt & Pärt, 2008; Kokko et al., 2004; Öst et al., 2011). Further, Dale, Lunde, and Steifetten (2005) predicted that habitat fragmentation may be an important factor promoting breeding dispersal. Flying squirrels live in very fragmented landscape (Santangeli et al., 2013; Selonen & Hanski, 2012), but we did not find a correlation between forest patch size and breeding movements.

A factor affecting breeding movements in flying squirrels is the mating system of the species. Male flying squirrels move between different females that live in separate territories (Selonen et al., 2013). Thus, males come to the nest site of a female, which may decrease reasons for females to enhance mating opportunities with breeding dispersal. In contrast, in mating systems where males aim to dominate mating opportunities of females, like in large ungulates, females may perform breeding movements with an aim to enhance reproductive success (Debeffe et al., 2014; Marjamäki, Contasti, Coulson, & McLoughlin, 2013). We made no such observations in our study, and radiotelemetry studies indicate that females do not make excursions out of their home range during mating season (Hanski et al., 2000; Selonen et al., 2013). In male flying squirrels, separating breeding dispersal from the normal movement to locate females is complicated due to the large home ranges of males (Hanski et al., 2000; Selonen et al., 2013). Evidently, some of the observed moves were long enough to potentially present breeding dispersal. That is, the moves may have led to a permanent change in home‐range location. However, the only factor related, albeit weakly, to breeding movements of males in our study was the male population size. In addition, contrary our prediction, a high population size of males decreased male breeding movements (for similar results, see Bond & Wolff, 1999).

The flying squirrel mating system may have elements of both scramble competition (Selonen et al., 2013), in which the mating success for males depends on search effort to locate females (Lane, Boutin, Gunn, & Coltman, 2009), and of a female defense system (Selonen et al., 2013), where mating opportunities for males depend on an individual's position in the male dominance hierarchy (Andersson, 1994; Koprowski, 2007). In addition, pairs of male and female flying squirrels often nest communally before the breeding season (Selonen et al., 2014). Thus, the pattern observed here for breeding movements of males may suggest that an increased male population size may enhance competition between males and lead to situations where males stay with a particular female and move less to locate new potential mates. However, the relationship was weak and may not have major effect on general movement patterns of males. In addition, it seems likely that the search effort for females has an important role in determining male mating success in a species with territorial females dispersed in space (Ims, 1988; Lane et al., 2009).

Availability of nest boxes was the only variable linked to breeding movements of females in our study. This pattern was driven by small‐scale movements, so that the possibility for a female to make short‐distance nest site changes depended on whether or not there were several nest boxes within a female territory. In fact, when we added a variable for whether or not a female moved out from the used forest patch in the model, then availability of nest boxes did not explain female breeding movements (results not shown). For males, nest‐box availability did not explain observed movement distances. Thus, the effect of box availability only had minor effects on observed breeding movement distances (see Section 2 for structure of our study area). Some of the measured environmental variables were themselves estimates (population size and food availability) and likely were influenced by measurement error. For this reason, and because we did not study home‐range‐level movement patterns, it is clear that factors driving small‐scale movements pattern in flying squirrels require further study (but see e.g., Selonen et al., 2001, 2013). However, for the current analysis, the main point is that no evidence could be detected that any of the measured environmental variables seem likely candidate for driving breeding dispersal in flying squirrels. Observed movement distances were obviously shorter than those observed in radio‐telemetry studies, but to a surprisingly little extent. For example, male home ranges were around 30 ha in our study areas (R. Wistbacka and P. Reunanen unpublished data, see also Hanski et al., 2000), which is comparable to the distance of nest site changes in this study. In addition, the observed natal dispersal distances were likely more underestimated than breeding movements in the current study.

Predators and parasites are two possible drivers of breeding movements not included in our study. For example, it is possible that predators in some cases induce breeding movements of flying squirrels, but we expect that inclusion of predators to our analysis would not have changed our main conclusions. The reason for this is that predator densities are low in northern latitudes and hunting territories of individuals are large (Saurola, 2008). Large hunting territories mean that it is not easy to move out from the hunting range of, for example, an Ural owl individual (*Strix uralensis*; the main predator of flying squirrels, Selonen, Sulkava, Sulkava, Sulkava, & Korpimäki, 2010). Anecdotal evidence also suggests that flying squirrels do not very easily change nest sites when a predator is present within the territory. In a radio‐telemetry study, a Ural owl individual was observed following movements of flying squirrels while these moved in and out of nest boxes (V. Selonen and I. K. Hanski, personal observation), but none of the flying squirrels in the area (*n* = 11) changed nest sites, although some individuals were killed by the Ural owl. Thus, we believe that it is unlikely that predators are at large extent driving breeding dispersal in our study areas, although some changes in movement patterns of individuals may occur when predators are present. Instead, fleas, the main parasite of flying squirrels, may increase breeding time nest changes, similar as observed, for example, for badgers, *Meles meles* (Butler & Roper, 1996). After the weaning of offspring, abandoned litter nests are often full of fleas (own observation), and it seems likely that avoidance of fleas is one potential driver of nest site changes observed in this study, in particular in adult females. However, these movements likely remain within a home range of an individual.

Natal philopatry was linked to territory vacancy after the apparent death of a mother. This is in line with earlier observations that territory vacancy is an important determinant of the settlement of natal dispersers in squirrels (Larsen & Boutin, 1994; Wauters & Dhondt, 1993; Wauters et al., 1995). However, territory bequeathal did not play a role in the behavior of flying squirrels. Earlier, Cockburn (1988) listed several rodent species that possibly bequeath territories for offspring, but later it was concluded that the evidence for this behavior was in many cases inadequate (Lambin, 1997). Price and Boutin (1993) concluded that territory bequeathal by mothers is more likely to occur when (1) juveniles are more able to defend the natal territory than to compete elsewhere for vacant territories; (2) having a territory is crucial for overwinter survival; and (3) in competition over territories, adults are better than juveniles. These terms could fit the case of flying squirrels, but no bequeathal occurred.

Understanding dispersal is central, as inadequate knowledge of movement ecology is still a major knowledge gap, for example, in conservation management (Driscoll et al., 2014). Our study supports the view that natal dispersal is the process governing gene flow and population spread. This emphasizes the importance of research on natal dispersal behavior, although it is a process difficult to study, as abandonment of the natal range is often a once in a lifetime process, usually occurring quickly during a short time period (Kokko & López‐Sepulcre, 2006). Our results also give an example of a forest rodent whose breeding movements are not related to variation in resource availability, habitat quality, or territory bequeathal. The movements of adult flying squirrels are related to the mating system, whereas the responsibility for redistributing individuals within and between populations is left for the next generation. We predict that the same might also be the case in other species in which males move between territories of females and individuals depend on nesting resources, which limits the possibility for territory shifts.

## Data Accessibility

The data set supporting the results of this article is available in the Eurasian Chronicle of Nature (formerly European Boreal Forest Biodiversity, EBFB) database repository, https://www.earthcape.com/. All applicable international, national, and/or institutional guidelines for the care and use of animals were followed.

## Conflict of Interest

None declared.
